# Disgust, Sadness, and Appraisal: Disgusted Consumers Dislike Food More Than Sad Ones

**DOI:** 10.3389/fpsyg.2018.00076

**Published:** 2018-02-06

**Authors:** Kosuke Motoki, Motoaki Sugiura

**Affiliations:** ^1^Department of Human Brain Science, Institute of Development, Aging and Cancer, Tohoku University, Sendai, Japan; ^2^Japan Society for the Promotion of Science, Tokyo, Japan

**Keywords:** food, disgust, sadness, appraisal-tendency framework, consumer preference

## Abstract

According to the affect-as-information framework, consumers base judgments on their feelings. Disgust is associated with two kinds of appraisal: one in which the consumer avoids and distances him/herself immediately from the object concerned, and another in which the consumer is disgusted due to contamination and impurities within the environment. The first instance indicates that disgust can decrease a consumer’s preference for a product, regardless of its category. In contrast, the second case suggests that a product’s degree of depreciation is greater in products vulnerable to contamination, such as foods. However, it remains largely unknown how incidental disgust affects product preferences in accordance with the two appraisal-related goals. The present research investigates how incidental disgust (as opposed to sadness, an equally valenced but distinct emotion of appraisal) influences consumer preferences for products with or without a risk of contamination. Twenty-four participants repeatedly judged foods or household products after seeing an emotional image (conveying disgust, sadness, or neutrality). Foods and household products are the two representative product categories in grocery stores, but only foods are associated with a risk of contamination. The results showed that incidental disgust led to negative evaluations of both types of products; however, compared to sadness, incidental disgust demonstrated a stronger negative effect on preference for foods than household products. These findings elucidate that disgust and the appraisal of contamination specifically devalue foods, and broaden the application of the appraisal-information framework in consumer settings.

## Introduction

Consumer behaviors are influenced by incidental emotions. That is, emotions that emerge while shopping, including reactions to advertising, products, prices, interactions with workers and friends, and memories, affect appraisals of products. According to the affect-as-information framework, affect provides information, and consumers attend to their feelings as sources of information while making judgments (Schwarz, 2011). Indeed, it is undeniable that many consumer decisions are made under emotionally charged conditions.

### Valence-Congruent Influences on Evaluations of Goods

Incidental affect has valence-congruent influences on evaluations of products when consumers use their emotions as sources of information for judgments. Most extant research in this domain has examined the influence of emotional valence (positive or negative) on consumer behaviors (Han et al., 2007; Cohen et al., 2008; Achar et al., 2016). According to the affect-as-information framework, positive emotional states are generally interpreted as indicative of liking, whereas negative emotional states are generally interpreted as indicative of disliking (Schwarz, 2011). Thus, it has been assumed that people who feel positive emotions increase their valuations of goods (Sherman et al., 1997), whereas people who feel negative ones decrease them (Axelrod, 1963).

### Incidental Emotions and Appraisal Tendencies

The mechanisms underpinning the use of affect as information extend the impact of appraisals involved with specific emotions beyond the influence of their emotional valence. Indeed, activating the appraisal leads to the corresponding subsequent consumer behaviors (Frijda et al., 1989; Smith and Lazarus, 1993; Han et al., 2007; Cohen et al., 2008; Achar et al., 2016), which seems to apply to certain types of goods.

Incidental specific emotions influence product preferences in accordance with appraisal-related goals. For example, anxiety is associated with appraisals characterized by uncertainty and a lack of control, and is likely to trigger a goal of reducing uncertainty, leading to risk avoidance behavior (Raghunathan et al., 2006). A study found that incidental anxiety led to greater depreciation of a new medication (uncertainty risk) than did sadness (Raghunathan et al., 2006). Moreover, guilt/shame, which is associated with feelings of distress from personal failure or transgressions, is likely to trigger the goal of reducing mindless or unethical behaviors (Arli et al., 2016). Moreover, incidental guilt/shame (by retrieving memories of overeating) elicits stronger negative emotions in relation to junk foods and images of mindless eating behaviors than to other foods (salad and burnt foods) (Piqueras-Fiszman and Jaeger, 2016). These studies indicate that discrete emotions correspond to specific appraisals and therefore shape subsequent product preferences in accordance with their respective functional goals.

### Incidental Emotions and Multiple Appraisal Tendencies

Discrete emotions are associated with several appraisals potentially influencing product preferences in accordance with each appraisal-related goal. Although specific emotions involve several appraisal dimensions beyond valence (e.g., anticipated effort, certainty, attentional activity, and self-other responsibility) (Smith and Ellsworth, 1985), previous studies have investigated how one appraisal among many can influence preferences for the appraisal-related products (Raghunathan et al., 2006; Piqueras-Fiszman and Jaeger, 2016). Consumer goods can be categorized into different groups (e.g., foods and household products), which may be associated with differential appraisals, linked to a specific emotion (e.g., foods associated with a risk for contamination). Understanding how several appraisals associated with an emotion variously influence preferences for the appraisal-related product can broaden the application of the affect-as-information framework in consumer settings. However, the issue remains uninvestigated. The present study addressed the issue by systematically investigating the influence of an emotion (disgust) on preferences of products linked differently to the two appraisal-related goals in comparison with an equally negative-valenced emotion (sadness).

### Disgust, Sadness, and Grocery Store Settings

Disgust and sadness seem to be common feelings that arise during shopping in grocery stores. Indeed, consumers encounter many products that may elicit disgust (e.g., trash bags, cat litter, and diapers) (The Food Institute, 2004) and sadness (sad music, advertisements, and cues that trigger associations of products with sad movies) (Goldberg and Gorn, 1987; Mitterschiffthaler et al., 2007). Grocery stores, generally, have two types of product: foods and household products. For example, consumers view and value chicken, cheese, potatoes, cake, kitchen sponges, toothbrushes, headache medicine, tissues and so on in grocery stores. However, it remains unknown how disgust and sadness influence preferences for the two representative categories in grocery stores.

### Disgust and the Two Appraisal Dimensions

Disgust may decrease product preferences more than sadness, regardless of the product type. Disgust involves appraisals associated with a strong impulse to avoid and distance oneself immediately from the object (Lerner et al., 2004), while sadness is interpreted as being indicative of loss and helplessness and is accompanied by an implicit tendency to alleviate its effect by seeking rewarding experiences (Raghunathan and Pham, 1999; Frijda, 2005; Lench et al., 2011). The appraisal associated with disgust indicates that consumers who feel disgusted devalue products through avoidance and by keeping a distance, while the appraisal of sadness indicates that consumers who feel sad may evaluate items as rewarding. Actually, previous studies have shown that incidental disgust decreases preferences for consumer goods and foods (Argo et al., 2006; Morales and Fitzsimons, 2007). Additionally, it has been shown that incidental sadness leads to increased consumption of foods viewed as indulgent (Garg et al., 2007), and an increased value for consumer goods mediated by an elevated sense of helplessness (Garg and Lerner, 2013). Together, it could be hypothesized that incidental disgust (as opposed to sadness) decreases preferences for goods to the same degree, regardless of the product type.

H1: Incidental disgust (vs. sadness) will decrease preferences for goods to the same degree, regardless of product type.

Disgust involves another appraisal, based on evolutionary perspectives, which indicates that devaluation by incidental disgust (as opposed to sadness) would be greater in products vulnerable to contamination, namely food. Evolutionary perspectives suggest that disgust is interpreted as being indicative of contamination and impurity within one’s environment (Griskevicius and Kenrick, 2013). Following an interpretation of a feeling evoked by a given food item as disgust, that item may be evaluated as contaminated. The appraisal indicates that the degree of devaluation by incidental disgust (as opposed to sadness) is greater in products susceptible to infectious risk, such as food.

H2: Following incidental disgust (as opposed to sadness), food items will be more devalued than household products.

### The Present Study

On the basis of the appraisal-tendency framework, we examined how incidental disgust influences consumer preferences for products in accordance with disgust-specific appraisals. Disgust involves two appraisals, which might differently affect product preference according to the risk of contamination. However, whether the disgust-contamination association influences consumer preferences differently in comparison with an equally valenced, but distinct, emotion of appraisal (sadness) remains uninvestigated.

To this end, we investigated how incidental disgust and sadness influence consumer preferences for categories of products with or without the potential of consumers perceiving a risk of contamination (foods or household products). In this study, 24 participants repeatedly reported their preferences for foods or household products after seeing an emotional image conveying disgust, sadness, or neutrality. Given one appraisal associated with disgust (defined as avoiding and distancing oneself immediately from the object), incidental disgust will devalue products to the same degree, regardless of product type, which is not the case for sadness. The data revealed a main effect of disgust but no significant interaction of sadness/disgust with foods/household products on consumer preferences. Given the other appraisal associated with disgust (marked by contamination and impurity in one’s environment), incidental disgust will devalue foods more than household products, as compared to sadness. The data revealed a significant interaction of sadness/disgust with foods/household products in relation to consumer preferences. Specifically, incidental disgust (as opposed to sadness) could devalue food more than household products.

## Materials and Methods

### Preliminary Study to Select Stimuli

We recruited 19 university students (7 females, *M*_age_ = 20.95 ± 2.17) from a bulletin board or mailing list for university students to select the products and emotional stimuli used in this study. This study was approved by the ethical committee of the School of Medicine at Tohoku University. Written informed consent was obtained from each subject, and the experiment was conducted in accordance with the Declaration of Helsinki. All participants were Asian (Japanese). The stimuli used in the preliminary study were obtained from the International Affective Picture System (IAPS) (Lang et al., 2008) or the Internet. The 19 participants evaluated foods and emotional images for selecting stimuli.

### Selecting Stimuli for Foods and Household Products

To select the stimuli representing products, the 19 participants (the same individuals who selected food images) rated their preferences for 154 foods and 122 household products on a 7-point Likert scale ranging from 1 (do not like very much) to 7 (like very much). Three sets of images of 20 foods (*M*_foodAgroup_ = 5.84, *M*_foodBgroup_ = 5.83, *M*_foodCgroup_ = 5.86) and 20 household products (*M*_householdAgroup_ = 4.84, *M*_householdBgroup_ = 4.82, *M*_householdCgroup_ = 4.84) were allocated to three emotion conditions (disgust/sadness/neutrality). The mean preference for foods was 5.85 ± 0.32, and the preferences for the three groups of food stimuli did not differ [*F*(2,57) = 0.040, *p* = 0.961, $\eta_{p}^{2}$ = 0.001]. The mean preference for household products was 4.83 ± 0.63, and the preferences for the three groups of household stimuli did not differ [*F*(2,57) = 0.003, *p* = 0.997, $\eta_{p}^{2}$ = 0.0001]. Even when comparing the sets of food and household products, with maximum differences (food C - household products B), with ones with minimum differences (food B - household products C), we did not find a significant difference [*M*_foodCandhouseholdB_ = 5.34, *M*_foodBandhouseholdC_ = 5.34; 95% CI (-0.30, 0.31), *t*(78) = 0.04, *p* = 0.966, *d* = 0.14]. The results showed that all combinations of foods and household products were equivalent for their preferences across participants. The three groups of foods and household products were randomized across participants for the three emotional conditions. The food images used in the main study included burgers, dumplings, fried foods, meat, noodles, pasta, pizza, plain rice, rice dishes, salad, seafood, soup, stew, and sweets. The household product images used in the main study included antiseptic solution, cleaning gloves, deodorizer, detergent, masks, medicine, pest exterminators, sanitizers, soap, sponges, sticking plaster, tissues, and toothpaste.

### Selecting Stimuli for Sadness, Disgust, and Neutral Emotions

To select the emotional stimuli, the 19 participants were each presented once with 243 affectively laden images and rated the extent to which they felt each specific emotion (anger, sadness, disgust, and fear), a general negative emotion, and arousal on a 7-point Likert scale from 1 (not at all) to 7 (very much). We calculated the mean ratings of each affectively laden image among participants, and then selected 10 images evoking disgust, 10 evoking sadness, and 10 evoking neutrality for allocation to the three emotional conditions (disgust/sadness/neutrality).

Images evoking disgust were rated as more disgusting (*M*_disgust_ = 6.22 ± 0.38) than sad [*M*_sadness_ = 3.32 ± 0.67, 95% CI (2.39, 3.41), *t*(18) = 11.87, *p* < 0.001, *d* = 5.31] or neutral [*M*_neutral_ = 1.08 ± 0.09, 95% CI (4.87, 5.38), *t*(18) = 41.94, *p* < 0.001, *d* = 18.76] images. Sad images garnered higher sadness ratings (*M*_sadness_ = 5.37 ± 0.20) than disgusting [*M*_disgust_ = 3.47 ± 0.63, 95% CI (1.46, 2.33), *t*(18) = 9.09, *p* < 0.001, *d* = 4.06] or neutral ratings [*M*_neutral_ = 1.09 ± 0.08, 95% CI (1.78, 2.67), *t*(18) = 63.38, *p* < 0.001, *d* = 28.34]. Images evoking disgust and sadness did not differ in terms of the negative emotion [*M*_disgust_ = 5.48 ± 0.25 vs. *M*_sadness_ = 5.42 ± 0.26, 95% CI (-0.19, 0.30), *t*(18) = 0.50, *p* = 0.624, *d* = 0.22] or arousal [*M_disgust_* = 3.95 ± 0.40 vs. *M*_sadness_ = 3.91 ± 0.37, 95% CI (-0.32, 0.41), *t*(18) = 0.27, *p* = 0.788, *d* = 0.12] that they elicited. Images evoking disgust and sadness garnered higher ratings for the negative emotions [*M*_disgust_ = 5.48 ± 0.25 vs. *M*_neutral_ = 1.25 ± 0.14, 95% CI (4.03, 4.42), *t*(18) = 45.81, *p* < 0.001, *d* = 20.49; *M*_sadness_ = 5.42 ± 0.26 vs. *M*_neutral_ = 1.25 ± 0.14, 95% CI (3.97, 4.37), *t*(18) = 43.70, *p* < 0.001, *d* = 19.54] and [arousal *M*_disgust_ = 3.95 ± 0.40 vs. *M*_neutral_ = 1.26 ± 0.17, 95% CI (2.39, 3.00), *t*(18) = 13.71, *p* < 0.001, *d* = 8.31; *M*_disgust_ = 3.91 ± 0.37 vs. *M*_neutral_ = 1.26 ± 0.17, *t*(18) = 19.55, 95% CI (2.36, 2.90), *p* < 0.001, *d* = 8.74] that they elicited than did neutral images.

### Participants

Participants (*n* = 25) were recruited from a bulletin board or mailing list for university students. This study was approved by the ethical committee of the School of Medicine at Tohoku University. Written informed consent was obtained from each subject, and the experiment was conducted in accordance with the Declaration of Helsinki. A power analysis was not conducted because there were no prior data concerning the emotional manipulation (passive viewing of photos) with consumer preferences. To ensure sufficient power for the detection of most effects, it has been suggested that, between subjects, at least 20 observations per condition are required (Simmons et al., 2011). Our design is within-subject. This offers a substantial boost in statistical power in comparison to the between-subjects design (Greenwald, 1976). There were 25 participants in our study, which may have enough power to confidently detect effects. Data from one participant were omitted due to missing values, and data from the remaining 24 participants (10 females, *M*_age_ = 21.38 ± 1.44) were used for all analyses. All participants were Asian (Japanese).

### Task Design

This task included three emotional conditions (disgust, sadness, and neutrality) and two types of product (foods and household products). A 3 (emotion: disgust, sadness, and neutrality) × 2 (product: foods and household products) within-subject factors was implemented, with both emotions and products being repeated. All participants experienced all combinations of emotions (disgust, sadness, and neutrality) and products (foods and household products).

**FIGURE 1:**
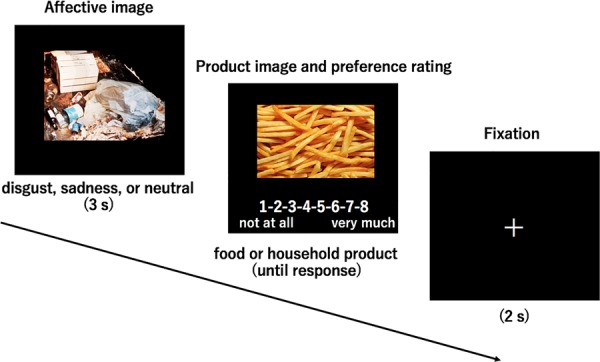
First, participants viewed affectively laden images (evoking disgust, sadness, or neutrality). After that, they viewed an image of a product (food or household product) and rated their preferences.

During the task, participants evaluated preferences for foods or household products after seeing the irrelevant and incidental emotional images (disgust, sadness, or neutral) (**Figure 1**). Combinations of a product image (foods/household products) and an emotional image (disgust, sadness, or neutral) were presented in a random order. Each emotional image was presented four times: twice before evaluating foods and household products, respectively. The number of trials was 120, and participants had a rest period after the 60th trial.

Each trial proceeded as follows. First, participants viewed affectively laden images (evoking disgust, sadness, or neutrality) on a screen for 3 s. After that, they viewed an image of a product (food or household product) and rated their preferences for the latter on a scale from 1 to 8 (1 = do not like very much, 8 = like very much).

The trial then concluded with a fixation cross that appeared for 2 s. Ten disgust-, sadness-, and neutrality-eliciting images were presented twice before the images of food products as well as before the images of household products were presented. The same images were presented before both types of product.

After completing the main task, participants rated the extent to which they felt disgust and sadness in response to each image using an 8-point Likert scale ranging from 1 (not at all) to 8 (very much).

## Results

### Induction of Incidental Emotions

The images that were selected to induce feelings of disgust did, indeed, evoke stronger feelings of disgust (*M*_disgust_ = 7.45 ± 0.58) than did those selected to induce sadness [*M*_sadness_ = 3.98 ± 1.54; 95% CI (2.88, 4.06), *t*(23) = 12.14, *p* < 0.001, *d* = 2.59] or neutrality [*M*_neutral_ = 1.32 ± 0.53; 95% CI (5.82, 6.43), *t*(23) = 41.52, *p* < 0.001, *d* = 11.03]. Likewise, the images selected to induce sadness induced stronger feelings of sadness (*M*_sadness_ = 6.73 ± 1.73) than did those selected to induce disgust [*M*_disgust_ 4.55 ± 1.48; 95% CI (1.52, 2.84), *t*(23) = 1.48, *p* < 0.001, *d* = 2.06] or neutrality [*M*_neutral_ = 1.48 ± 0.93; 95% CI (4.72, 5.78), *t*(23) = 41.52, *p* < 0.001, *d* = 5.17]. These results show that we successfully induced the intended emotions.

### Effects of Incidental Disgust, Sadness, and Neutrality on Preferences for Foods and Household Products

We conducted repeated-measures analysis of variance (ANOVA; 3 emotions: sadness, disgust, and neutral by 2 types of product: foods/household products). If Mauchly’s sphericity assumption was violated, the Greenhouse-Geisser adjustment was used to correct sphericity by altering the degrees of freedom using a correction coefficient epsilon. Bootstrap re-sampling with 2000 replications was used to deliver 95% CIs of the effect size ($\eta_{p}^{2}$).

**FIGURE 2:**
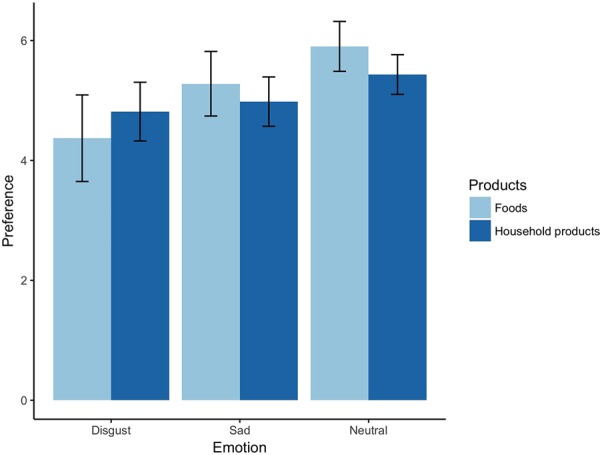
Preferences for foods depreciated more following feelings of incidental disgust than following feelings of incidental sadness, whereas this was not the case for household products. Error bars represent standard deviation.

The ANOVA showed a significant main effect of emotions [Greenhouse-Geisser correction: *F*(1.26,29.06) = 26.37, *p* < 0.001, $\eta_{p}^{2}$ = 0.53, bootstrapped 95% CI (0.38, 0.64)], but no significant main effects of product types [*F*(1,23) = 0.27, *p* = 0.606, $\eta_{p}^{2}$ = 0.01, bootstrapped 95% CI (0.00, 0.10)]. The analysis also showed significant interactions of emotion by type of product [Greenhouse-Geisser correction: *F*(1.37,31.59) = 5.93, *p* = 0.013, $\eta_{p}^{2}$ = 0.21, bootstrapped 95% CI (0.02, 0.48)], such that the incidental emotions differentially affected consumer preferences according to the type of product (**Figure 2**).

### Disgust Depreciated Foods (vs. Household Products) Preferences More Than Sadness

For *post hoc* analysis, we ran ANOVA (2 emotions: disgust/sadness by 2 types of product: foods/household products) because the interaction term in the analysis was the main variable in this study. The ANOVA revealed a main effect of emotion, *F*(1,23) = 21.92, *p* < 0.001, $\eta_{p}^{2}$ = 0.50, bootstrapped 95% CI (0.26, 0.66), such that preferences for products decreased more following incidental disgust than incidental sadness. However, there was not a main effect of type of product, *F*(1,23) = 0.08, *p* = 0.774, $\eta_{p}^{2}$ = 0.004, bootstrapped 95% CI (0.00, 0.03). The analysis also showed significant interactions of emotion by type of product, *F*(1,23) = 13.11, *p* = 0.001, $\eta_{p}^{2}$ = 0.36, bootstrapped 95% CI (0.13, 0.57), such that the disgust and sadness differentially affected consumer preferences according to the type of product.

Furthermore, we estimated differences in preferences between product types by subtracting the ratings assigned to foods from those assigned to household products (preferences for foods – ones for household products) under the incidental disgust/sadness condition. We conducted a paired *t*-test to compare the differences in preferences between product types, and between disgust and sadness. The results showed that food preferences (as opposed to household product preferences) were lower following disgust than following sadness [*M*_disgust_ = -0.44 ± 1.52 vs. *M*_sadness_ = 0.30 ± 1.11; *t*(23) = -3.62, 95% CI (-1.17, -0.32), *p* = 0.001, *d* = 0.56].

Given that the effects of emotions differ in men and women (Kring and Gordon, 1998), we performed the same analyses with sex as a covariate, and the same results were observed in all analyses.

### Do Sadness and Disgust Increase or Decrease Food/Household Product Preferences Compared with Neutrality?

Next, we investigated whether sadness and disgust increase or decrease product preferences as compared to neutral emotions. We estimated a “disgust effect” and a “sadness effect” by subtracting the ratings assigned to foods and household products under the incidental disgust/sadness condition from those assigned to them under the neutral condition. Positive disgust/sadness effects reflect that disgust/sadness reduce preferences for products, whereas negative effects reflect that disgust/sadness increase preferences for products.

### Effects of Disgust on Preferences for Foods and Household Products

To investigate whether disgust increases or decreases food/household product preferences, we conducted a one-sample *t*-test. The results showed that the “disgust effect” was significant for both foods [*M*_disgust_food_ = 1.53 ± 1.67; 95% CI (0.83, 2.24), *t*(23) = 4.49, *p* < 0.001, *d* = 0.92] and household products [*M*_disgust_household_ = 0.62 ± 0.80; 95% CI (0.28, 0.96), *t*(23) = 3.78, *p* = 0.001, *d* = 0.77], indicating that incidental disgust decreased preferences for products regardless of type, which supports the valence-congruent hypothesis.

To investigate the differential effects of disgust on consumer preferences for different types of product, we compared the effects of disgust on foods and household products. As expected, the results showed that disgust decreased preferences for foods (*M*_disgust_food_ = 1.53 ± 1.67) more strongly than it decreased preferences for household products [*M*_disgust_household_ = 0.62 ± 0.80; 95% CI (-0.36, 0.70), *t*(23) = 2.52, *p* = 0.019, *d* = 0.70].

### Effects of Sadness on Preferences for Foods and Household Products

To investigate whether sadness increases or decreases food/household product preferences, we conducted a one-sample *t*-test. In contrast to our hypothesis, the “sadness effect” was significant for foods, indicating that sadness was associated with a more pronounced *decrease* in preferences for foods than was neutrality [*M*_sadness_food_ = 0.62 ± 0.97; 95% CI (0.21, 1.03), *t*(23) = 3.15, *p* = 0.005, *d* = 0.64]. Sadness also decreased preferences for household products [*M*_sadness_household_ = 0.45 ± 0.71; 95% CI (0.15, 0.75), *t*(23) = 3.11, *p* = 0.005, *d* = 0.63]. The results indicate that incidental sadness decreased preferences for all types of product and did so in a valence-congruent manner.

Comparison of the effects of sadness on foods and household products did not reveal significant differences [*M*_sadness_food_ = 0.62 ± 0.97 vs. *M*_sadness_household_ = 0.45 ± 0.71; 95% CI (-0.36, 0.70), *t*(23) = 0.67, *p* = 0.509, *d* = 0.20].

Together, these results supported H2: Following incidental disgust (as opposed to. sadness), food items will be more devalued than household products.

## Discussion

According to the affect-as-information framework, consumers make judgments based on their feelings. Although the specific appraisals associated with unique emotions influence consumer preferences, disgust involves two kinds of appraisal, which might influence preferences differently for products with or without a risk of contamination. However, the issue remains uninvestigated. Using the two representative product categories in grocery stores as stimuli, the present research investigated how incidental disgust (as opposed to sadness) influences consumer preferences for foods (with a risk of contamination) and household products (without a risk of contamination). The results showed that incidental disgust led to negative evaluations of both types of product, and that it had a stronger negative effect on preferences for foods than on household products, which was not the case for sadness. These findings elucidate the idea that disgust and appraisals of contamination specifically devalue foods and broaden the application of the appraisal-information framework in consumer settings.

The findings contributed, theoretically, to the appraisal-of-information framework in that they identify the influence of the two appraisals involved with disgust (as opposed to sadness) in product preferences. Although disgust decreased consumer preferences more than sadness in accordance with one of the appraisals associated with disgust (defined as avoiding and distancing oneself immediately from the object), the degree of devaluation was product-category-dependent. Supporting the other appraisal associated with disgust (marked by contamination and impurity in one’s environment) (Griskevicius and Kenrick, 2013), incidental disgust (as opposed to sadness) devalued food more than household products. The second appraisal is derived from evolutionary motives and the tendency to avoid disease (Griskevicius and Kenrick, 2013). The latter is likely to be triggered by disgusting cues of potential pathogens, such as sneezing, coughing, foul odors, skin lesions, and other abnormalities. Feelings of incidental disgust may motivate an individual to avoid disease and to perceive food, specifically, as potentially contaminated, which in turn would decrease preferences for food more than preferences for household products. Taken together, the results indicate that evolutionary-based appraisals associated with disgust have a different influence on appraisal-related products, such as foods.

The influences of specific emotions on preferences coexist in valence-congruent as well as appraisal-dependent ways. Consistent with the valence-congruence hypothesis, negatively valenced emotions, disgust and sadness, decreased preferences for all types of product. Additionally, the degree to which such preferences decreased preferences was moderated by differences in the informational characteristics of appraisals and the types of product. Disgust decreased preferences for foods more than sadness and did so in an appraisal-dependent way. Thus, these analyses expanded the theoretical implications of the effects of incidental emotions on consumer judgments. The results did not exclude the influence of either appraisal or typology, instead suggesting that specific emotional influences on preferences for products coexist in valence-congruent as well as appraisal-dependent ways.

In contrast to previous findings, we found that incidental sadness decreased preferences for all types of product. This discrepancy may be due to cultural differences between the participants in this study (East Asians) and those in previous research (Westerners). Although previous research has shown that experimentally induced sadness led consumers to pay more money for products than did experimentally induced neutrality and disgust (Lerner et al., 2004), this “sad premium” has been observed when consumers feel sad while focusing on themselves (Cryder et al., 2008). East Asians, including Japanese individuals (like the participants in this study), are less self-focused than Westerners during experiences of sadness (Mesquita and Leu, 2007). Cultural differences between Westerners and East Asians with regard to the self-focus during experiences of sadness may explain the discrepant results. Thus, sadness may increase preferences for products among Westerners due to their high level of self-focus, but it may have the opposite effect in East Asians owing to their lower level of self-focus.

The present study revealed that passively viewing images was enough to affect consumer preferences for appraisal-dependent goods. Previous studies used various methods to induce emotions (movies, pictures, recall of emotional events, reading) and a meta-analysis suggested that the effect size of movie clips was greater than that of other methods (Angie et al., 2011). However, as consumers seem to experience emotions most frequently (and repeatedly) in response to passively viewing images (e.g., packages, products, pictorial advertisements) (Holbrook and Batra, 1987; Desmet, 2004; Reimann et al., 2010), it is important to determine whether the passive viewing of pictures changes preferences for appraisal-dependent goods. Taken together, our results showed that repeatedly presented emotional images, which are probably similar to those consumers experience during shopping, were enough to shift consumer preferences for the informational goods depicted.

### Practical Contributions

These results can contribute to management decisions about store layout and design. Although negative emotions decrease consumer preferences in general, managers should be most careful about the locations of foods and cues eliciting disgust. Many cues elicit feelings of disgust, including those associated with sanitary goods, products touched by others, and items with ripped tags or messy displays. The cues that elicit disgust should be removed before consumers touch food, because these feelings may substantially depreciate consumer preferences for such food. Alternatively, managers should separate food sections from items or ads that could, potentially, arouse disgust. Taken together, the results indicate that managers should lay out their stores in such a way that consumers do not simultaneously encounter foods and items or ads associated with feelings of disgust.

### Limitation and Future Study

Although we divided products into two broad categories (foods and household products), there are more, detailed, product categories that may correspond with the appraisals associated with disgust and sadness, respectively. In the case of food and disgust, certain foods such as meats, raw fish, and shellfish spoil more quickly and have a high risk of contamination compared to other foods. Incidental disgust may depreciate these perishable foods more than others. In the case of foods and sadness, sweets and junk food seem more rewarding than healthier options such as salad. Incidental sadness may increase preferences for sweets and junk food but not for salad. This kind of research is needed to clarify, in more detail, the mechanisms of interplay between incidental emotions and product preferences.

Finally, this study demonstrated that incidental disgust influences preferences for appraisal-related products. Although disgust depreciates product preferences, regardless of product type, the degree of devaluation of food by disgust (as opposed to sadness) was greater than that of household products. The findings showed that incidental disgust influences consumer preferences for products with or without a risk for contamination, and deepen our understanding of the affect-as-information framework, especially with regard to disgust and products in grocery stores.

## Ethics Statement

This study was approved by School of Medicine, Tohoku University.

## Author Contributions

Conception and design of the study: KM and MS. Analysis and interpretation of the data: KM. Collection and assembly of the data: KM. Drafting of the article: KM and MS. Final approval of the article: KM and MS.

## Conflict of Interest Statement

The authors declare that the research was conducted in the absence of any commercial or financial relationships that could be construed as a potential conflict of interest.
